# Accuracy benchmark of the GeneMind GenoLab M sequencing platform for WGS and WES analysis

**DOI:** 10.1186/s12864-022-08775-3

**Published:** 2022-07-22

**Authors:** Chaoyang Li, Xue Fan, Xin Guo, Yongfeng Liu, Miao Wang, Xiao Chao Zhao, Ping Wu, Qin Yan, Lei Sun

**Affiliations:** 1 GeneMind Biosciences Company Limited, Shenzhen, China; 2 The Third People’s Hospital of Longgang District, Shenzhen, China; 3 The department of Pediatric, Longgang District Maternity&Child Healthcare Hospital of Shenzhen City, Shenzhen, China

**Keywords:** GenoLab M, NovaSeq 6000, Nextseq 550, WGS, WES, NA12878

## Abstract

**Background:**

GenoLab M is a recently developed next-generation sequencing (NGS) platform from GeneMind Biosciences. To establish the performance of GenoLab M, we present the first report to benchmark and compare the WGS and WES sequencing data of the GenoLab M sequencer to NovaSeq 6000 and NextSeq 550 platform in various types of analysis. For WGS, thirty-fold sequencing from Illumina NovaSeq platform and processed by GATK pipeline is currently considered as the golden standard. Thus this dataset is generated as a benchmark reference in this study.

**Results:**

GenoLab M showed an average of 94.62% of Q20 percentage for base quality, while the NovaSeq was slightly higher at 96.97%. However, GenoLab M outperformed NovaSeq or NextSeq at a duplication rate, suggesting more usable data after deduplication. For WGS short variant calling, GenoLab M showed significant accuracy improvement over the same depth dataset from NovaSeq, and reached similar accuracy to NovaSeq 33X dataset with 22x depth. For 100X WES, the F-score and Precision in GenoLab M were higher than NovaSeq or NextSeq, especially for InDel calling.

**Conclusions:**

GenoLab M is a promising NGS platform for high-performance WGS and WES applications. For WGS, 22X depth in the GenoLab M sequencing platform offers a cost-effective alternative to the current mainstream 33X depth on Illumina.

## Background

The past 15 years have witnessed a new era in DNA sequencing technologies [1], starting from the release of the Roche 454 sequencer, which opened the door to next-generation sequencing (NGS) [2]. Compared to Sanger sequencing technology [3], NGS has remarkably higher throughput and reduced costs [1]. As technology upgrades and iterates, NGS technologies have dramatically decreased the cost of human whole genome sequencing (WGS) and whole-exome sequencing (WES). As a result, the rapid development of technology leads to brilliant achievements in WGS projects such as the 1000 genome project [4], the HapMap project [5], and extensive cohort studies worldwide. WGS and WES have been and are being widely performed to discover disease-associated genes and identify driver mutations in hereditary tumors [6–8]. It lays the foundations for the understanding of how mutated genes affect disease phenotype and the further interpretation of pathogenic mechanisms [6–8].

Since the completion of the Human Genome Project in 2003, various sequencing platforms have been developed: Roche 454, Illumina series (GA, HiSeq, Miseq, NextSeq, NovaSeq, etc.) [9], MGI (BGISEQ-500, MGISEQ2000, DNBSEQ-T7) [10], Ion Torrent [11], and GenapSys [12]. Benefiting from continued technology development and product commercialization, Illumina’s sequencing by synthesis (SBS) based sequencers have dominated the sequencing market for a long time. In 2016, NextSeq 550 was released as mid-throughput desktop sequencing instrument, which can be applied in many fields, including transcriptome sequencing, targeted sequencing, WES, metagenomics sequencing, and genotyping. In June 2017, NovaSeq 6000 was launched, which incorporates Illumina’s SBS chemistry and two-color optics. Combined with patterned flow cell technology and reversible terminator-based method [10], it can produce 6 TB of sequencing data in a single run at a cost of approximately 10 USD/GB [13]. As NGS applications expand in various research areas and clinical settings, there is an unmet demand to develop a novel NGS platform that is accurate, flexible, and cost-efficient for applications.

In October 2020, GeneMind Biosciences Company Limited (GeneMind) launched a new sequencing instrument (GenoLab M) based on their previous work on single molecule sequencer GenoCare™ [14]. The GenoLab M sequencer employs SBS techniques and reversible termination approaches [15]. In 2021, the first study using GenoLab M was published [15], revealing that the GenoLab M is a promising sequencing platform for transcriptomics and LncRNA studies in animal, plant, and human with comparable performance but a lower cost compared to NovaSeq 6000. However, the performance of the GenoLab M platform in other application areas has not yet been released, especially in WGS and WES.

In 2014, Genome in a Bottle (GIAB) published A golden standard genotype dataset (including reference sample NA12878), providing a resource for comparison of variants calling pipelines [16]. Recently, several studies used the GIAB variant dataset for comparisons among different variants callers or sequencing platforms [17–20]. Generally, data depth of WGS and WES were above 30 fold and 100 fold [13, 18, 21–23]. Early in the history of WGS, the field converged around the concept that 30-fold represents a “high quality” genome with the ideal trade-off of accuracy and cost. Together with Genome Analysis Tool kit (GATK) [24] as the best practice analysis pipeline [25], this depth concept has become deeply ingrained in the community mindset, even when the sequencing and analysis fields have evolved rapidly. It is well recognized that GATK works well with dominated Illumina data, but is not yet proven on other sequencing platforms. Also, 30-fold data in WGS is potentially redundant, not only on the cost of sequencing but also the analysis computation and storage costs. There are quite a few previously published lower depth WGS studies, such as a large group WGS project of Icelanders in 2015 with a median sequencing depth was 20X [26]. In 2018, Anna Supernat et al., have compared three variant callers (DeepVariant [27], GATK, and SpeedSeq [27]) for WGS reference sample sequenced at different depths (10X, 15X, and 30X). It was observed that the F-Scores obtained by DeepVariant at 15X were comparable to SpeedSeq and GATK at 30X. Yifan Jiang et al., found that the optimal sequencing depth for whole genome re-sequencing in pigs was 10X, an ideal practical depth for achieving plateau coverage and discovering accurate variants with greater than 99% genome coverage [28]. With all these preliminary supporting studies and the emerging sequencing and analysis technologies with improved accuracy, a lower sequencing depth than 30X may be considered as the current best practice.

This study obtained both WES and WGS datasets of the NA12878 standard sample generated from multiple sequencing platforms, including NextSeq 550, NovaSeq 6000, and GenoLab M. On the analysis part, two pipelines were chosen: Sentieon DNAscope pipeline, a machine learning (ML) based variant calling workflow (https://github.com/Sentieon/sentieon-dnascope-ml), and DNAseq workflow, which is an accelerated GATK re-implementation [29]. We compared WGS performance in GenoLab M with 22X data and NovaSeq 6000 with 33X data.

## Method

### Samples preparation and sequencing

We ordered 50 μg NA12878 cell line genomic DNA from Sequanta Technologies Co., Ltd. After quality control, in brief, the genomic DNA was constructed as Illumina WES via SureSelect Human All Exon V8 kit (Agilent Technologies Inc.) and WGS library via TruSeq Nano DNA library kit (Illumina, Inc.). Subsequently, one ug DNA to was fragmented by Covaris E220 to 100–250 bp for WES, and to 350–450 bp for WGS. Then, end of each DNA fragment was repaired and an A base was added to the 3’end to form a sticky end, and then the Illumina adapter was ligated to both ends of DNA fragments. PCR amplification was applied to each sample after ligation. While WGS libraries were completed, the WES libraries went through additional steps, including SureSelect Human All Exon V8 capture, PCR amplification and purification.

WES library was split and loaded into GenoLab M and NextSeq 550 or NovaSeq 6000 for 150 bp paired-end sequencing. And WGS library was sequenced on GenoLab M and Novaseq. For GenoLab M, the sequence process was referred to reference 15. Briefly, the library is denatured to single-stranded and surface-based amplified on the flow cell. Then, the amplified DNA colonies are hybridized to a sequencing primer. Next, Fluorescence-dye labeled nucleotides and a polymerase are added to start the sequencing cycle. In each cycle, the nucleotides’ terminator structure ensures only one nucleotide is incorporated. Four-color fluorescence signals from the labels are collected by a scanning optical system, and then the terminator structure is cleaved. Finally, the fluorescence image data are then combined and color-corrected, sequencing quality score are assigned to each base to produce the final fastq file.

### Reads mapping and bam processing

Secondary analysis was performed via Sentieon software v 202,112.01 [30], a complete suite of tools that can be used to process raw reads to variant calling result. Raw reads were aligned to the hg38 (https://ftp-trace.ncbi.nlm.nih.gov/giab/ftp/release/references/GRCh38/) by “Sentieon BWA” and sorting was done by the “sort” utility tool. BAM files were then adjusted by Samtools v1.10 to the desired depth for later analysis and comparison, specifically 22X and 33X for the WGS dataset, and 100X for the WES dataset. Quality metrics were generated from these BAM files by Sentieon QC tools. Next, “LocusCollector” and “Dedup” tools were used to mark duplicate reads, to prepare the BAM files for variant calling step.

### Running DNAseq (GATK re-implementation) and DNAscope

The Sentieon DNAseq pipeline is a re-implementation of the GATK best practice pipeline, returning identical results at a much higher speed [29]. DNAseq is typically five to ten fold faster than GATK pipeline on the same generic CPU platform. Therefore here in this study, we ran DNAseq pipeline and treated the result the same as the data from GATK pipeline. Deduped BAM files were firstly processed by “QualCal” tool to conduct base quality score recalibration, and variants were called by “Haplotyper” tool to provide the matching result of GATK. VQSR was not performed because we do not believe this extra step will improve overall variant calling accuracy [31].

Deduped BAM files were directly input into DNAscope pipeline, as BQSR step is not needed here. DNAscope variant caller first generated candidate variants, filtered in the next step. GenoLab M ML model was applied on both variant generation and filtering steps. DNAscope is designed as a successor to GATK HaplotypeCaller, as it uniquely combines the well-validated methods from haplotype-based variant callers with ML to achieve improved accuracy. The candidate variants calling comprises three parts: active region detection, local haplotype assembly, and read-likelihood calculation (Pair-HMM). Later the variant candidates with rich annotations are passed to a ML model for variant genotyping, leading to improvements in both variant calling and genotyping accuracy.

The GenoLab M model for DNAscope was constructed during this project using several WGS and WES datasets sequenced from reference samples. Due to the limited training dataset, separated WGS and WES models were trained. The training was performed across all chromosomes with the exception of chromosome 20. It should be noted that none of the evaluated datasets was used during training.

### Variant accuracy evaluation

All VCF files generated from DNAseq or DNAscope pipelines were taken as input for accuracy evaluation. They were compared against the NIST truth set v4.2.1 using hap.py v0.3.14 with RTGtools vcfeval v3.10.1 as the variant comparison engine [32] to calculate an F-score as a representation of accuracy. Stratification region files v2.0 were downloaded from GIAB project and used for stratification analysis [33]. We calculate Precision, Recall and F-score referred to [17], and the details were as follows:

1. Ture Positive (TP): variants called by a variant caller in high confident regions as the same genotype as the gold standard data.
2. Ture Negative (TN): reference alleles in high confident regions other than gold standard variants.
3. False Positive (FP): variants called by a variant caller in high confident regions but not as the same genotype as the gold standard data.
4. False Negative (FN): gold standard variants in high confident that were not called by a variant caller.
5. Precision: TP/(TP + FP), meaning positive predictive value, is the fraction of relevant instances among the retrieved instances.
6. Recall: TP/(TP + FN), meaning sensitivity, is the fraction of relevant instances that were retrieved.
7. F-score: 2* Precision*Recall/(Precision+Recall), is the harmonic mean of the precision and recall.

## Results

### NGS datasets summary

**Fig. 1:**
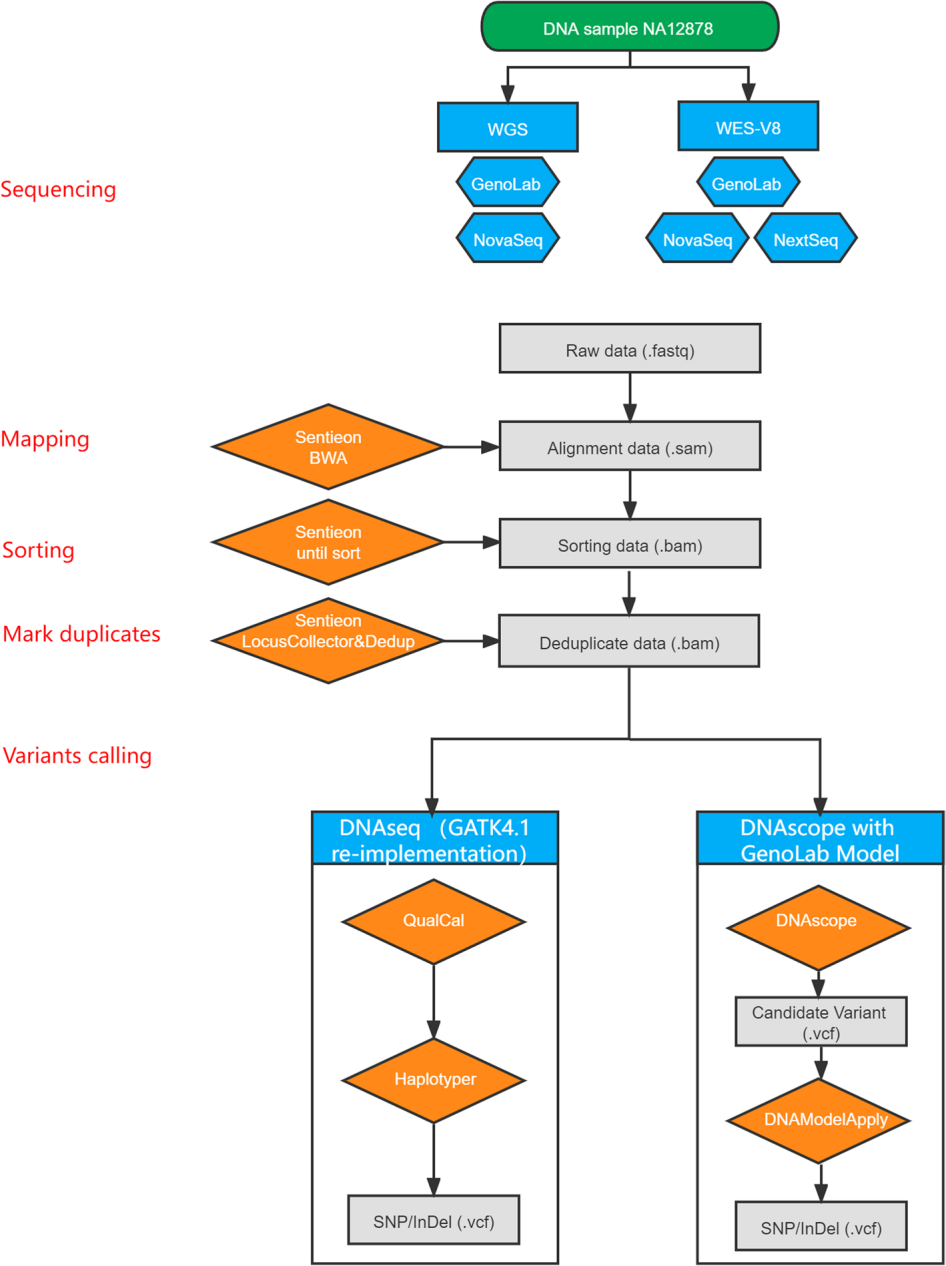
The flowchart of combinations using three sequencers and two variant calling pipelines for germline variants. Key process for NGS data generation and analysis were shown on the left. Squares in the flowchart represent data files, and rhombus indicate processes. NovaSeq means NovaSeq 6000, NextSeq means NextSeq 550

To avoid biased results by different sample prep and library construction processes, we used the same WGS or WES library. In total, there are three WES and two WGS datasets obtained from GenoLab M, and NovaSeq 6000 or NextSeq 550 (Fig. 1), and the dataset were subsampled to an average of 100X in whole exome for WES and an average of 22X in whole genome for WGS to generate additional datasets for comparison. FASTQ and BAM quality statistics were calculated, as shown in Table 1. For the base quality (over Q20) base percentages, the GenoLab M showed an average of 94.62%, slightly lower than NovaSeq’s performance at 96.97%. While the duplication rate of GenoLab M outperformed NovaSeq or NextSeq, which was only half of NovaSeq’s duplication rate at the same sequencing depth. A lower duplication rate usually leads to higher data usage and less waste.

**Table 1:** Statistics of the multiple sequencing datasets in our study

Samples	Library Type	Sequencing Platform	Read (M)	Bases (Gb)	Duplication rate (%)	>Q20	>Q30	Alignment rate (%)	Mean coverage (X)	%_bases_above_15x
GL_WGS_22	WGS	GenoLab M	442.77	66.42	1.73%	95.35%	88.26%	99.88%	22.39	81.30%
GL_WGS_33	WGS	GenoLab M	662.66	99.40	1.93%	95.22%	87.99%	99.88%	33.50	93.90%
NA_WGS_22	WGS	NovaSeq 6000	424.9	63.73	3.57%	95.92%	90.05%	99.64%	21.37	87.30%
NA_WGS_33	WGS	NovaSeq 6000	655.83	98.38	5.32%	95.92%	90.05%	99.64%	32.99	97.70%
GL_WES_100	WES Agilent V8	GenoLab M	41.87	6.28	6.00%	93.95%	84.71%	99.95%	112.42	98.00%
GL_WES_raw	WES Agilent V8	GenoLab M	70.36	10.55	9.71%	93.95%	84.71%	99.95%	188.90	99.00%
NA_WES_100	WES Agilent V8	NovaSeq 6000	39.35	5.90	14.85%	98.01%	94.05%	99.95%	107.72	99.30%
NA_WES_raw	WES Agilent V8	NovaSeq 6000	81.16	12.17	26.78%	98.01%	94.05%	99.95%	222.19	99.60%
NT_WES_100	WES Agilent V8	NextSeq 550	37.54	5.56	5.67%	86.62%	79.06%	99.83%	101.13	99.30%
NT_WES_raw	WES Agilent V8	NextSeq 550	131.76	19.50	17.54%	86.61%	79.06%	99.83%	354.92	99.60%

### The performance of 22× WGS data in GenoLab M

**Fig. 2:**
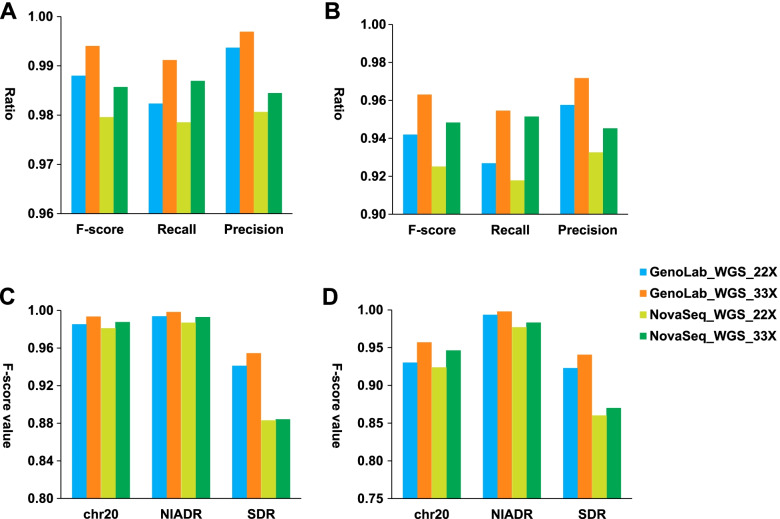
Comparison of variants calling performances in GenoLab M and NovaSeq 6000 from 33X and 22X coverage of the NA12878 sample. A SNP and B InDel on whole genome, C SNP and D InDel F-score on stratification region. Precision, positive predictive value, is the fraction of relevant instances among the retrieved instances, Recall, sensitivity is the fraction of relevant instances that were retrieved. F-score is the harmonic mean of the precision and recall, chr 20 means chromosome 20, NIADR means Not in all Difficult Regions, SDR means Segmental Duplications Regions

Subsequently, we compared the WGS SNP&InDel calling accuracy of GenoLab M and NovaSeq with analysis algorithms adapted to each sequencer at 22X and 33X depth. As shown in Fig. 2A&B, the F-score, Recall, and Precision of SNP and InDel from 33X WGS were higher than 22X WGS from the same sequencing platform. At the same depth, GenoLab M showed higher recall and precision in SNP and InDel calling than NovaSeq. Interestingly, 22X WGS from GenoLab M had similar performance in SNP, and a slight advantage in InDel, compared to 33X WGS from NovaSeq. GenoLab M’s analysis ML model could be part of the reason. The characteristics of the sequencing data are also likely to contribute to the difference. In addition, stratification comparison was performed including Chromosome 20 (chr20), which was not included in any of DNAscope’s model training dataset; Segmental duplications region (SDR); and “Not in all Difficult Regions” (NIADR). As displayed in Fig. 2C&D, stratification comparison was similar to the whole genome, especially in SDR, 22X GenoLab M dataset reached better performance (F-score of 0.941 and 0.923, respectively) in SNP and InDel calling compared to 33X NovaSeq dataset (F-scores 0.884 and 0.870, respectively).

**Fig. 3:**
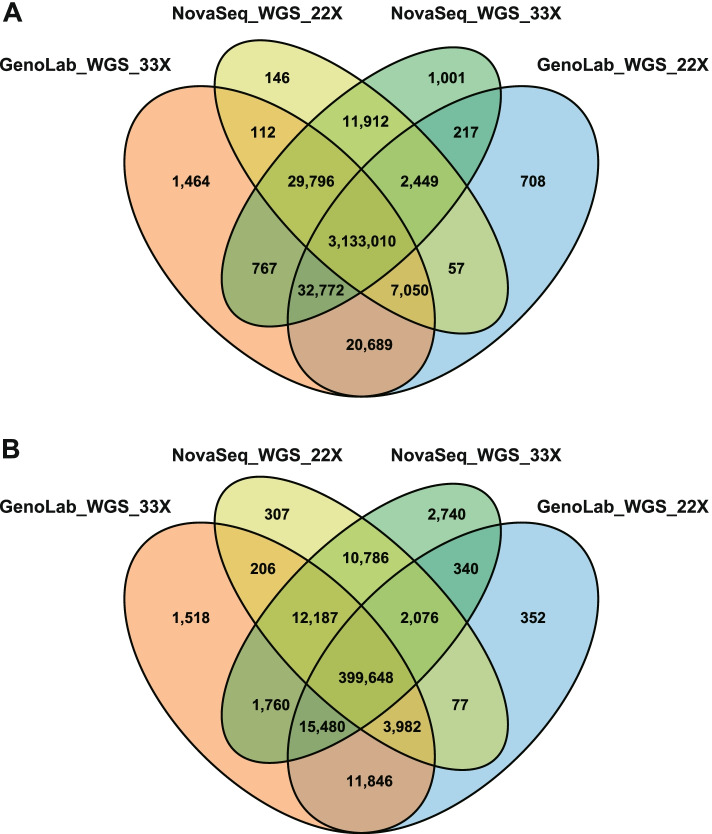
Venn diagram of variants calling performances in WGS datasets. A SNP and B InDel

The variant calling results of two platforms at 22X or 33X depth were filtered using GIAB NA12878 truth vcf file. The distribution of the after-filter variants representing concordance of each dataset was shown in Venn diagrams (SNP, Fig. 3A and InDel, Fig. 3B). For common sets of variants, the proportion of SNP (96.27%, 3,133,010) was significantly higher than that of InDel (85.45%, 399,648). Besides, 22X WGS from GenoLab M (98.24 and 92.75%) showed indistinguishable SNP detection and slightly inferior InDel, compared with 33X data from NovaSeq (98.70 and 95.15%).

### Variants calling performance in WES datasets

**Fig. 4:**
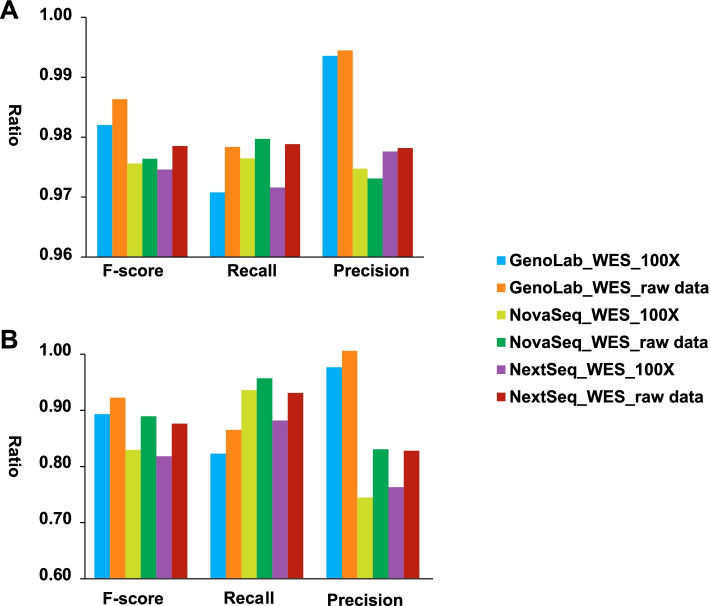
Comparison of variants calling performances in six WES datasets..A SNP and B InDel. Precision, positive predictive value, is the fraction of relevant instances among the retrieved instances, Recall, sensitivity is the fraction of relevant instances that were retrieved. F-score is the harmonic mean of the precision and recall

Three WES datasets at their raw sequencing depth and three more datasets subsampled to 100X were generated for WES performance assessment. As expected, SNP and InDel F-score, Recall, and Precision of the subsampled datasets dropped from their original depth (Fig. 4). At 100X, the F-score and Precision in GenoLab M were higher than NovaSeq or NextSeq, while the Recall in GenoLab M was slightly lower.

**Fig. 5:**
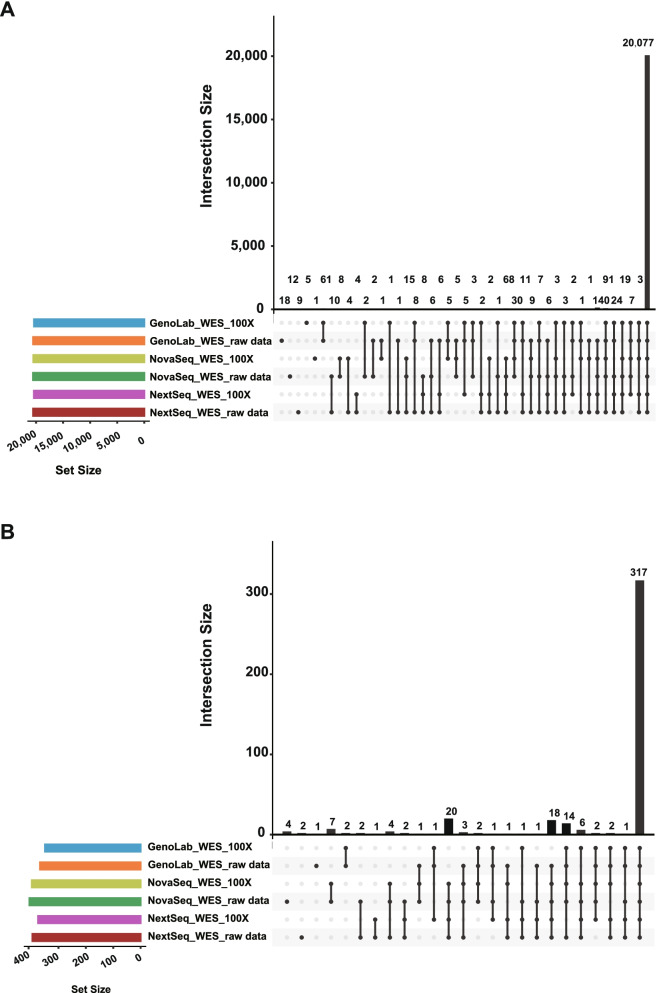
Upset diagram of variant Calling results of all combinations in WES datasets. A SNP and B InDel

Same as with WGS concordance analysis, the variant calling results of six WES datasets were filtered by reference truth, and concordance was shown in Fig. 5. All six datasets jointly identified 20,707 SNPs and 425 InDels, which were more than 97% of the truth variants’ amount, with the majority shared among all six datasets. For InDel, 100X depth in all platforms has no specific number, compared with raw data, while, for SNP, GenoLab M and NovaSeq have a small number of mutation detection. Overall, at 100X depth, GenoLab M (20,371) displayed comparable recall in SNP detection compared with NovaSeq (20,490) or NextSeq (20,388), and slightly inferior in InDel detection.

## Discussion

In the past 10 years, with the development of NGS sequencers by companies such as Illumina, MGI, and Ion Torrent, the application of WES or WGS to identify variants of the human genome became accessible for the public and even individuals. To further expand the accessibility, various variants calling pipelines have been developed to adapt each of these sequencing platforms, introduced by published benchmark studies. For WGS, 30-fold represents a “high quality” genome, and GATK is one popular bioinformatics analysis tool.

In this study, WES and WGS datasets of the NA12878 standard sample were generated from NextSeq 550, NovaSeq 6000, and GenoLab M. We measured the base quality (Q20&Q30), duplication rate, and the average sequencing depth of each dataset. Since GenoLab M is a new sequencing platform, GenoLab M’s ML model for DNAscope was constructed using several WGS and WES datasets generated from reference samples. For Illumina platforms, GATK pipeline analysis was performed. For Q20 percentages, the GenoLab M showed an average of 94.62%, and the NovaSeq 6000 was 96.97%, with a slight preponderance towards better result. At the same time, the duplication rate of GenoLab M was only half of NovaSeq 6000 under the same sequencing depth (Table 1).

Analysis observed that 22X GenoLab M WGS showed higher accuracy than 22X NovaSeq accuracy and reached a similar performance of 33X NovaSeq (Fig. 2A&B). Both low duplication sequencing and GenoLab M analysis ML model contribute to the variant calling accuracy. Here we believe GenoLab M offers a cost-effective alternative to the NovaSeq 6000 platform with less depth (22X) and similar data quality for human resequencing applications. GenoLab M’s lower duplication rate may lead to better data efficiency. The human genome shows a complex pattern of highly identical, interspersed segmental duplication, also known as SDR [34, 35]. This region poses particular challenges for gene annotation because:

1. Enriched in assembly gaps [36];
2. More prone to copy number polymorphism among individuals [37];
3. Different paralogs are difficult to distinguish because of their high sequence identity [38].

The existence of SDR predisposes humans to large-scale rearrangements due to unequal crossing-over leading to genomic instability associated with neurodevelopmental delay and autism [39]. The demonstrated accuracy advantages of GenoLab M sequencing platform in the SDR of the human genome may be suitable to NGS projects on neurodegeneration disease and autism.

In WES analysis, recall of GenoLab M was still lower than NovaSeq or NextSeq at the same sequencing depth, which serves as a development target for us. To improve overall variant calling accuracy, more GenoLab M reference datasets are required to assemble a larger training set for future DNAscope model training. Also, the collection and sequencing of more clinical or scientific samples will further help GeneMind R&D to improve sequencing instruments’ performance, such as increasing the Quality value (Q20&Q30) and throughput.

## Conclusions

For WGS, 22X in GeneMind sequencing platform showed a similar performance to 33X depth in Illumina NovaSeq 6000, which offers an effective alternative. And 100X WES of GenoLab M showed similar or superior performance to Illumina platforms at the same depth, which also has application prospects in WES.

## Data Availability

The bam files of WGS and WES are available in CNGB Sequence Archive (https://db.cngb.org/cnsa/) under project accession number CNP0002694.
